# Bioactive’s Characterization, Biological Activities, and In Silico Studies of Red Onion (*Allium cepa* L.) Skin Extracts

**DOI:** 10.3390/plants10112330

**Published:** 2021-10-28

**Authors:** Florina Stoica, Iuliana Aprodu, Elena Enachi, Nicoleta Stănciuc, Nina Nicoleta Condurache, Denisa Eglantina Duță, Gabriela Elena Bahrim, Gabriela Râpeanu

**Affiliations:** 1 Faculty of Food Science and Engineering, Dunarea de Jos University of Galati, 111 Domneasca Street, 800201 Galati, Romania; florina.stoica@ugal.ro (F.S.); iuliana.aprodu@ugal.ro (I.A.); elena.ionita@ugal.ro (E.E.); nicoleta.stanciuc@ugal.ro (N.S.); nina.condurache@ugal.ro (N.N.C.); gabriela.bahrim@ugal.ro (G.E.B.); 2 National Institute of Research & Development for Food Bioresources—IBA Bucharest, 6 Dinu Vintila Street, 021102 Bucharest, Romania; denisa.duta@bioresurse.ro

**Keywords:** red onion skins, anthocyanins, antioxidant activity, thermal stability, molecular modeling, biological activity

## Abstract

This study aimed to investigate the thermal stability and biological activities of the phytochemicals from the red onion skins extract, which are a rich source of anthocyanins. Eight anthocyanins were identified in the extract by high-performance liquid chromatography, the most abundant ones being cyanidin 3-*O*-laminaribioside and cyanidin 3-*O*-(6″-malonoyl-laminaribioside). The study also involved the assessment of the thermal degradation kinetics of anthocyanins and antioxidant activity in the 75–155 °C temperature range. The thermal degradation kinetics was described using the first-order kinetics model. In terms of thermal stability, increasing the temperature resulted in lower half-life values (t_1/2_) and higher degradation rate constant values (k) for both anthocyanins and antioxidant activity. The thermodynamic parameters revealed that the phytochemicals’ degradation is a non-spontaneous and endothermic reaction. Furthermore, the inhibitory effect of the extract was investigated against the enzymes affiliated with metabolic syndrome, oxidative stress, and inflammatory process diseases. Thus, we also demonstrated that the red onion skins extract exerted inhibitory activity on α-glucosidase, α-amylase, lipase, and lipoxygenase. Considering the high content of bioactives and various biological properties, the red onion skins extract is suitable for multiple applications.

## 1. Introduction

The onions (*Allium cepa* L.) are among the world’s oldest cultivated vegetables that are easily adaptable in a wide range of environments. The onion is used either fresh or processed, as an essential ingredient in many recipes, being accepted by almost all cultures [1]. Onions are important for both feeding and medicinal purposes. In the last years, the consumption of onion has expanded significantly due to its flavor and health benefits. These beneficial properties are related to the high content of organosulfur and phenolic compounds [2]. However, the onions’ processing generates large quantities of wastes, mainly skins. For example, more than 500,000 tons of onion wastes are generated annually in the European Union and need useful management [3]. The onion wastes, particularly the non-edible dry skins, can be a potential source of bioactive compounds, mainly anthocyanins, and also can be processed and reused as bioactive food ingredients [4].

The red onion skins are rich in anthocyanins (cyanidin and peonidin derivatives) and flavonols (quercetin derivatives) [5]. The anthocyanins are a group of naturally occurring pigments derived from plant raw materials. They have remarkable importance due to their dual role: as an integral part with sensory attributes (color) and as compounds with health benefits and disease prevention effects [6]. The health-promoting effects are related to the antioxidant, anti-proliferative, anti-inflammatory, and cardioprotective activities, helping in regulating the lipid metabolism and improving insulin resistance [7]. Various studies [8,9,10,11] have shown that the red onion skins can exhibit inhibitory activity against the enzymes connected to metabolic syndrome and oxidative stress. Color is also a major attribute having a high influence on the appearance and the acceptance of food products.

The thermal treatment process is among the most widely used preservation methods to expand the food shelf-life and to ensure food safety [12]. Depending on the product attributes and intended shelf-life, food processing may include the thermal treatment in the 50–150 °C temperature range [13]. The anthocyanins are highly unsteady and susceptible to degradation by extrinsic factors such as temperature, pH, oxygen, metal ions, light, enzymes, etc. [14]. Similarly, the phenolic compounds, as well as the antioxidant activity, can be degraded through heating. In this context, it can be predicted that industrial processing at different temperature-time combinations might cause changes in the levels of biologically active compounds. These changes lead to organoleptic and nutritional properties loss as well [13]. Due to their health benefits, there is a real need to preserve the content of anthocyanins, polyphenols, and antioxidant capacity in red onion skin extract. It is important as well to secure the optimal color and nutritional quality of food during thermal processing. Therefore, a precise understanding of the thermal degradation mechanisms based on mathematical models allows the optimization of industrial processing [15].

The purpose of the present study was to extract and characterize the anthocyanins found in the red onion skins. The thermostability, thermal degradation kinetics, and thermodynamic parameters associated with the total anthocyanins content (TAC) and antioxidant activity of the extract were investigated. Moreover, molecular modeling was used to simulate the thermal-dependent behavior of the main anthocyanin molecules from the onion skins. In addition, the biological activities of the extract were evaluated against α-glucosidase, α-amylase, pancreatin lipase, and lipoxygenase (LOX), enzymes associated with different diseases. Molecular docking tests were finally carried out to check the ability of the anthocyanins from the onion skins to recognize and interfere with the catalytic site of these enzymes.

## 2. Results and Discussion

### 2.1. Extraction and Characterization

The extraction was performed using the ultrasound-assisted extraction (UAE) method with aqueous ethanol 70% acidified with glacial acetic acid (in a 6:1 ratio, % (*v/v*)), and the red onion skins extract was characterized in terms of phytochemical content and antioxidant activity (Table 1). The anthocyanins are the compounds responsible for the red/purple color of red onion, are highly concentrated in the skins and the outer fleshy layers of red onion [16,17].

In our study, the extract presented an average TAC of 2.75 ± 0.15 mg C3G/g DW. Our results are in agreement with other studies reported by Ali et al. [18], Katsampa et al. [19], Viera et al. [15], and Makris [20]. Ali et al. [18] reported 2.25 mg C3G/g red onion skins anthocyanins to extract prepared with 0.01% HCl acidified ethanol. Katsampa et al. [19] found 2.09 mg C3G/g DW in the red onion skins extract obtained with the UAE method using 90% (*w/v*) aqueous glycerol as solvent. Viera et al. [15] reported a TAC of 227.7 ± 16.2 mg C3G/100 g DW using the conventional extraction method with aqueous ethanol 60% (*v/v*), after 30 min of extraction, while Makris [20] found a TAC of 183.85 mg C3G/100 g after 3.7 h of extraction with aqueous ethanol 60% (*v/v*).

The total polyphenols content (TPC) of the red onion skins extract was 172.17 ± 3.01 mg GAE/g DW. This value is in line with the ones reported by Viera et al. [15] who reported 188.4 ± 58.8 mg GAE/g DW for the red onion skins to extract obtained with aqueous ethanol 40% (*v/v*), after 60 min at 25 °C. Škerget et al. [21] reported a TPC of 114.86 ± 1.15 mg GAE/g of red onion skins to extract, after 5 h of extraction with aqueous ethanol 60% (*v/v*) at 25 °C. Higher values were reported by Yang et al. [22] who found a content of 233.40 ± 0.58 mg GAE/g of extract when using aqueous ethanol 70% (*v/v*) as a solvent, and Lee et al. [23] who acquired a TPC of 372.50 ± 6.85 mg GAE/g of extract obtained with aqueous ethanol 70% (*v/v*) at 60 °C for 3 h extraction.

The antioxidant activity of the red onion skins extract was determined by measuring the radical-scavenging activity on DPPH. After 30 min of reaction, the red onion skins extract showed antioxidant activity of 436.25 ± 3.51 mM TE/g DW. A similar antioxidant activity (490.54 ± 9.43 mM TE/g DW) for the ultrasounds treated red onion skins extract was determined by Prokopov et al. [24] using aqueous ethanol 70% (*v/v*). Viera et al. [15] determined the antioxidant activity of the red onion skins extract obtained by conventional extraction, and the highest value of 100.1 ± 4.9 μM TE/g DW was obtained with aqueous ethanol 60% (*v/v*) after 240 min at 25 °C. All these authors reported variable results for the phytochemical content of the red onion skin extract due to the differences regarding the phytochemical variability in raw material, environment, and extraction conditions.

### 2.2. Chromatographic Profile of the Anthocyanins from the Red Onion Skins Extract

The anthocyanins from the red onion skins extract were separated and identified at 520 nm using the HPLC technique. The identification of the anthocyanins was achieved based on the retention time (RT) and by comparison to the available standards.

**Figure 1:**
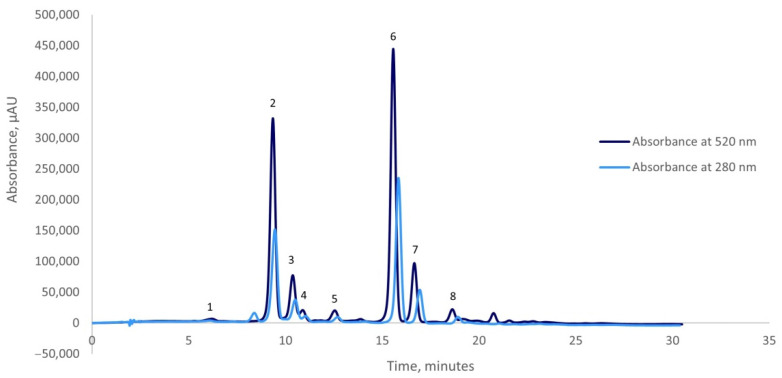
Chromatographic profile of the anthocyanins from the red onion skins extract obtained by ultrasounds-assisted extraction: Peak (1) cyanidin 3-*O*-glucoside; Peak (2) cyanidin 3-*O*-laminaribioside; Peak (3) cyanidin 3-*O*-(3″-malonylglucoside); Peak (4) peonidin 3-*O*-glucoside; Peak (5) cyanidin 3-*O*-(6″-malonylglucoside); Peak (6) cyanidin 3-*O*-(6″-malonyl-laminaribioside); Peak (7) peonidin 3-*O*-malonylglucoside and Peak (8) cyanidin 3-dimalonylaminaribioside.

The HPLC-DAD profile of the red onion skins extracts revealed the presence of eight compounds: cyanidin 3-*O*-glucoside, cyanidin 3-*O*-laminaribioside, cyanidin 3-*O*-(3″-malonylglucoside), peonidin 3-*O*-glucoside, cyanidin 3-*O*-(6″-malonylglucoside), cyanidin 3-*O*-(6″-malonyl-laminaribioside), peonidin 3-*O*-malonylglucoside, and cyanidin 3-dimalonylaminaribioside (Figure 1). Two major compounds were found in the red onion skins extract: cyanidin 3-*O*-laminaribioside with a concentration of 29.95%, and cyanidin 3-*O*-(6″-malonyl-laminaribioside) with a concentration of 38.93% of the total anthocyanins content. Peonidin 3-*O*-malonylglucoside, as the third major compound, constitutes 8.55% of the total anthocyanin content. Each of the other identified compounds contributed with a concentration lower than 8% of the total anthocyanins content. These observations comply with our previous study, in which the main anthocyanins found in the red onion skins extract were cyanidin 3-*O*-laminaribioside, cyanidin 3-*O*-(6″-malonyl-laminaribioside), and peonidin 3-*O*-malonylglucoside [25]. In general, our results are in agreement with the literature data [26], the most commonly reported anthocyanins in the red onion being cyanidin derivatives. Also, in the literature, the acetylated anthocyanins with malonic acid are the predominant pigments identified in the red onion chromatographic profile [16]. Donner et al. [27] also identified cyanidin 3-*O*-laminaribioside and cyanidin 3-*O*-(6″-malonyl-laminaribioside) in different red onion extracts, but in their study cyanidin 3-*O*-(6″-malonylglucoside) and cyanidin 3-*O*-glucoside were the major ones.

### 2.3. Heat Treatment

**Figure 2:**
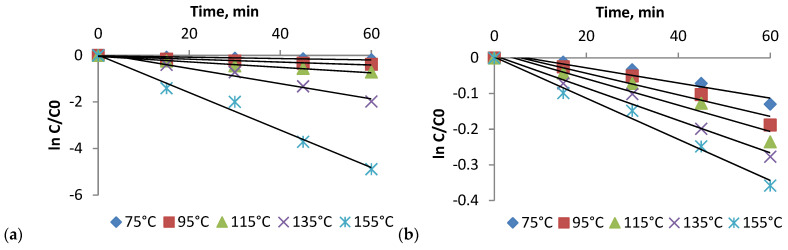
Thermal degradation kinetics of TAC (**a**) and antioxidant activity (**b**) of red onion skin extract that was treated at different temperatures (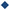 75 °C, 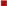 95 °C, 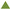 115 °C, 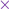 135 °C, 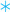 155 °C); C_0_ and C are the TAC or antioxidant activity before and after thermal treatment (mg/g dw).

The heat treatment results showed that increasing the temperature in the 75–155 °C range influenced the TAC. The decrease of TAC was observed as time and temperature increased (Figure 2a). In this study, the thermal degradation of red onion skins anthocyanins followed the first-order reaction kinetics (R^2^ > 0.9). These results are similar to those reported by Turturică et al. [28,29] and Oancea et al. [30].

Our results showed that the beginning of the degradation process occurred slowly at 75 °C and was more intense at 155 °C. Thus, the TAC in red onion skin extract presented a 7.60% decrease after 15 min of heating at 75 °C and 99.25% after 60 min of thermal treatment at 155 °C. Similar results were obtained in other studies performed on other plant matrices. For example, Turturică et al. [29] reported a severe reduction of the TAC from sweet cherry skins to extract, between 47% and 63% after 60 min of heat treatment at temperatures ranging from 90 °C to 120 °C. Oancea et al. [30] also reported that, after 120 min of thermal treatment at temperatures of 100 and 150 °C, the anthocyanins from sour cherry skins extract decreased by almost 42% and 96%, respectively. In plums (*Prunus domestica*) skins extract, the TAC degradation percentage after 20 min of thermal treatment at 70 °C and 110 °C was 47% and 91%, respectively [28].

The degradation was substantially slower at lower temperatures and faster at higher heating temperatures, indicating that time and temperature have a strong influence on anthocyanin stability, as expected [31]. The degradation of anthocyanins is mainly caused by oxidation, cleavage of covalent bonds, or enhanced oxidation reactions as a result of thermal processing. Probably during heat treatment, anthocyanins or their conjugated sugars are broken down into small molecules [31,32].

In the case of antioxidant activity, the first-order kinetic model was found to be the most suitable for representing the effect of thermal degradation (Figure 2). Figure 2b shows that the antioxidant activity of the red onion skin extract decreased throughout the heating temperature and time. However, as expected, the degradation was less pronounced when lower temperatures were used. The decrease of the antioxidant activity of the extract heat-treated for 60 min in the temperature domain 75–115 °C, ranged from 12.16% to 20.98%. The degradation process intensified when higher temperatures were applied; the extract lost 30.09% of the initial antioxidant activity after 60 min of heat treatment at 155 °C. Therefore, it can be appreciated that the antioxidant potential of the extract was maintained throughout the studied temperature range. Over the same temperature range, the antioxidant activity exhibited a lower decrease compared to TAC. The thermal degradation trend is rather similar to other antioxidant compounds like phenolics (flavonoids), phenolic acids, alk(en)yl cystein sulphoxides that in term presented a much better thermostability [33].

These results are in agreement with the results reported by Oancea et al. [30], and Turturică et al. [28,29]. Oancea et al. [30] found a decrease of 10–17% of the antioxidant activity of the sour cherry skins extract during the 60 min thermal treatment at 100–150 °C. In sweet cherries skins extract, heating caused an antioxidant activity decrease between 22–27% in the temperature range of 50–100 °C after 15 min. The decrease attained a maximum value of 44% after 60 min at 120 °C [29]. Turturică et al. [28] observed a reduction of the antioxidant activity of plums skins extract between 3–12% up to 5 min of heating at the temperature range of 70–90 °C. The rate of the antioxidant activity loss gradually increased with increasing the heating time, reaching a maximum value of 61% after 20 min at 110 °C.

In general, the thermal degradation of anthocyanins begins with the hydrolysis of sugar moieties. A careful analysis of the anthocyanin molecules heated by the in silico approach at temperatures resembling the experimental ones showed that planarity of the aromatic rings in the anthocyanidins structure changed with the intensity of the thermal treatment. Equilibrating the anthocyanin models at increasing temperatures from 75 to 155 °C resulted in more advanced tilting of the phenyl aromatic ring towards the benzopyrylium. Moreover, a slight increase of the C-O-C angle ensuring the connection between sugar and anthocyanidin was observed with the temperature; the C-O-C increased from 125.13° (at 75 °C) to 133.05° (at 155 °C) for the cyanidin 3-*O*-laminaribioside, and from 125.66° (at 75 °C) to 128.61° (at 155 °C) in case of cyanidin 3-*O*-(6″-malonyl-laminaribioside). In addition, the 8.5% stretching of the bonds involved in defining this angle supports the hypothesis of potential anthocyanins deglycosylation at high temperatures. The deglycosylation of anthocyanins results in the formation of anthocyanidins, which are further degraded in chalcones. The breakdown of chalcones generates the formation of phenolic acids and carboxaldehyde [34]. It has been supposed that the degradation products of anthocyanins display antioxidant properties [35]. Moreover, Sui and Zhou [36] showed negligible losses in the antioxidant capacity of aqueous solutions of anthocyanins after thermal treatment at a temperature range of 100–165 °C. The formation of other phenolic compounds by the anthocyanins’ degradation was suggested to be the reason for the nearly constant antioxidant capacity observed in the aqueous solutions.

### 2.4. Estimation of TAC and Antioxidant Activity Degradation Kinetic Parameters

The degradation of TAC and antioxidant activity of the red onion skins extract was further studied by analyzing the kinetic parameters. Table 2 presents the kinetic parameters: the kinetic rate constants (k), the half-life values (t_1/2_), D parameter values, z-values, and activation energy (Ea) values.

The kinetic rate constant allows the prediction of the TAC’s thermal degradation. The lower the k value, the higher the TAC stability. In our study, the k values ranged from (0.69 ± 0.08) × 10^−2^ min^−1^ at 75 °C to (18.65 ± 2.36) × 10^−2^ min^−1^ at 155 °C. It is clear from Table 2 that as the temperature increased, the k values also increased, indicating that a higher degradation occurred at higher processing temperatures. This finding points out that temperature strongly affects the stability of anthocyanins. Al-Qadri [37] analyzed the kinetic parameters of anthocyanins degradation in red onion skins extracts at different temperatures. The thermal degradation rate constants showed values of 66 × 10^−2^ h^−1^ at 100 °C, 25 × 10^−2^ h^−1^ at 70 °C, and 8.3 × 10^−2^ h^−1^ at 50 °C. Turturică et al. [29] reported k values ranging from (9.00 ± 2.58) × 10^−2^ min^−1^ at 70 °C to (10.87 ± 1.57) × 10^−2^ min^−1^ at 120 °C for TAC thermal degradation in sweet cherry skins extract. Oancea et al. [30] studied the TAC kinetics degradation during the heat treatment of sour cherries skins extract and reported the increase of the degradation constant rate with increasing temperature.

The D values decreased with increasing temperature, showing clear differences in the thermal sensitivity at different temperatures. At 75 °C, the D value was 333.33 ± 6.85 min, while a decrease of approximately 96.3% was observed by increasing the temperature to 155 °C.

The z parameter expresses the increase in temperature required to reduce D by 10%. The z-value reported in Table 2 asserts that the thermal resistance factors for several food characteristics, such as the color given by the targeted bioactives, are greater than the value needed for the spores or vegetative cells (z = 5–12 °C). Hence, the rates of the degradation of the color are much less temperature-sensitive [38]. Nayak et al. [39] reported a z-value of 47.9 °C for the purple potatoes anthocyanins’ degradation while the D parameter varied between 87.9–8.1 min after heat treatment in the temperature range of 100–150 °C. Peron et al. [12] studied the degradation of anthocyanins from grape and juçara extracts between 50 and 90 °C and suggested z-values of 23.2 °C in juçara extract and 24.4 °C in the “Italia” grape extract. Also, they reported D values between 1715—31 h, and 312—7 h respectively.

Another way to express the degradation of TAC is by using the t_1/2_ values. As shown in Table 2, it was noticed that as the temperature increased, the t_1/2_ values decreased consistently with faster reactions accompanied by higher k values. This trend is in good agreement with other studies from the literature [30,37,39]. Al-Qadri [37] previously reported that t_1/2_ values of anthocyanins in a red onion skins extract were 83.49 h at 50 °C and 10.5 h at 100 °C. Oancea et al. [30] studied the kinetics of anthocyanins thermal degradation in sour cherries skins extract in the temperature range of 100–160 °C for different heating periods and pointed out the high thermostability of anthocyanins. They reported the t_1/2_ ranging from 158.40 ± 5.77 min at 100 °C to 11.44 ± 0.54 min at 160 °C. Lower t_1/2_ values of 26.45 min at 100 °C and 2.42 min at 150 °C were registered by Nayak et al. [39], when characterizing the anthocyanins from the purple potato extract.

In general, the activation energy (Ea) is used to characterize the energy needed to reach the active state of a reaction [40]. The Ea for TAC degradation was calculated as 50.77 ± 1.71 kJ·mol^−1^. The Ea was similar to the one determined by Oancea et al. [30] for the sour cherries skins anthocyanins (Ea of 54.19 ± 5.88 kJ mol^−1^). In the study conducted by Al-Qadri [37], Ea values of 39.10 (kJ/mol) were reported after the thermal treatment of red onion skin extract. Turturică et al. [28] found a Ea of 36.42 ± 2.89 kJ mol^−1^ for the thermal degradation of anthocyanins in the plum skins extract.

The kinetics data of the antioxidant activity degradation from red onion skin extract during heating were shown in Table 2. The k values increased from (0.48 ± 0.27) to (1.33 ± 0.84) × 10^−2^ min^−1^ as the temperature increased. This trend indicates that the antioxidant activity rate of degradation varied with the temperature. Similarly, Turturică et al. [28] stated that the k values for the antioxidant activity thermal degradation in plums skin extract increased with increasing the temperature. Significantly higher k values were found in sweet cherries skins extract, ranging from (9.27 ± 1.76) × 10^−2^ min^−1^ at 70 °C to (10.65 ± 2.83) × 10^−2^ min^−1^ at 110 °C accordingly to Turturică et al. [29]. Lower degradation rates, ranging from (0.09 ± 0.01) × 10^−2^ min^−1^ at 100 °C to (8.12 ± 1.48) × 10^−2^ min^−1^ at 160 °C, were observed by Oancea et al. [30] in sour cherries skins extract.

The t_1/2_ of the antioxidant activity thermal degradation in red onion skins extract was 143.32 ± 2.42 min at 75 °C but decreased to 51.89 ± 1.46 min at 155 °C (Table 2). The results showed that the DPPH radical scavenging activity of the red onion skins extract was more stable at low-temperature treatment. In accordance, Turturică et al. [28] reported that the antioxidant activity thermal degradation of plum extracts also followed the first-order reaction and the t_1/2_ decreased from 173.28 to 33.00 min when the temperature increased from 70 to 110 °C. Lower t_1/2_ values were found in sweet cherries skins extract, ranging from 7.47 ± 0.71 at 70 °C to 6.50 ± 0.43 min at 110 °C [29].

The Ea for the degradation of the antioxidant activity within the temperature domain considered in the study was 15.13 ± 2.05 kJ/mol (Table 2). The low value of Ea means that the antioxidant activity of the red onion skin extract is less sensitive to temperature change. Turturică et al. [28] reported an Ea value of 47.22 ± 5.78 kJ·mol^−1^ for the skins of the plums to extract, while in another study [29], they reported an Ea value of 8.56 ± 1.42 kJ·mol^−1^ for the sweet cherries skins to extract.

### 2.5. Thermodynamic Parameters Calculation

To evaluate if the kinetic model used in this study is thermodynamically possible, the estimation of the thermodynamic parameters was performed. The physical and chemical phenomena of TAC and antioxidant activity degradation were determined through thermodynamic studies. Knowing the thermodynamic parameters allows a deeper understanding of the thermal degradation kinetics, to minimize the undesired degradation processes and optimize the quality of the food products [12]. Table 3 presents the activation enthalpy (ΔH), the Gibbs free energy (ΔG), and the activation entropy (ΔS) at 75, 95, 115, 135, and 155 °C.

The activation enthalpy (ΔH) is defined as a measure of the energy barrier that must be overcome by the reacting molecules. Moreover, it is associated with the bonds which are broken and reformed during the intermediate states resulting from the reactants [41,42]. The ΔH values of TAC ranged from 47.88 ± 0.62 to 47.21 ± 0.27 kJ/mol. The ΔH values of antioxidant activity ranged from 12.24 ± 0.16 to 11.57 ± 0.40 kJ/mol. The positive values reveal that the bioactives’ degradation was an endothermic reaction and prove that red onion skins’ phytochemicals degraded with increasing temperature.

The Gibbs free energy (ΔG) describes the equilibrium and spontaneity of the activated state and reactants [43]. The ΔG calculated for the TAC and antioxidant activity of red onion skins extract showed values ranging from 111.91 ± 1.37 to 126.65 ± 2.18 kJ/mol and 112.95 ± 3.53 to 136.03 ± 2.12 kJ/mol, respectively. From Table 3, it can also be seen that the ΔG values increased with the temperature due to the high degradation rate of TAC and antioxidant activity at higher temperatures. The positive values of ΔG indicate that the phytochemicals thermal degradation was a nonspontaneous reaction [41,44].

The activation entropy (ΔS) is defined as the disorder degree of molecules in the system and is usually related to the number of molecules with appropriate energy to react [12]. The ΔS values calculated in this study were all negative, varying from −184.02 ± 2.18 to −185.61 ± 1.56 J/(mol·K) for the TAC, and from −289.39 ± 1.41 to −290.79 ± 1.06 J/(mol·K) for the antioxidant activity. These values indicate that there might be lesser structural freedom of the intermediate state (complex) compared to the reactant, resulting in the presence of an entropy barrier in the system. Also, it confirms that thermal degradation is an irreversible process.

Not many studies present the values of thermodynamic parameters, even though these parameters are important in estimating the mechanism of thermal degradation. Al-Qadri [37] reported a decrease in the values of entropy and enthalpy with the increase of the free energy for the red onion skin extracts thermally treated in the 40–100 °C temperature range. Moreover, Turturică et al. [29] estimated the thermodynamic parameters at different temperatures (70−120 °C) for the thermal degradation of anthocyanins in sweet cherries skins extract. They also reported an increase in the ΔG and ΔH values over the temperature range.

### 2.6. In Vitro Enzyme Activity Inhibition 

The anthocyanins are phytochemicals of particular interest because of their potential health benefits [45]. The latter refers also to the benefits against metabolic syndrome (MetS) associated diseases. MetS is a clustering disorder comprising obesity, abnormal insulin and glucose metabolism, hypertension, disturbed blood lipids, pro-inflammatory state, and dyslipidemia [46]. Different enzymes like α-glucosidase, α-amylase, lipase, and LOX are involved in the appearance of these metabolic alterations. The most common clinical treatment in the management of MetS involves the prescription of enzyme inhibitors, such as acarbose for α-glucosidase and α-amylase, and orlistat for pancreatic lipase. However, the continuous intake of these drugs can cause adverse effects [47,48]. Thus, natural alternatives with fewer secondary effects are being currently studied.

The in vitro inhibitory effects of the red onion skins extract was assessed towards the enzymes associated with MetS: α-glucosidase, α-amylase, pancreatic lipase, and LOX. The inhibitory activity exerted by the biologically active compounds from the red onion skins was tested using three extract concentrations, namely 0.5, 1, and 5 μg/mL. Acarbose, orlistat, and quercetin were used as positive controls. The results were expressed as IC_50_ values (Table 4).

The red onion skins extract was active against α-amylase and α-glucosidase with IC_50_ values of 0.57 ± 0.16 μg/mL of extract for α-glucosidase, and 1.02 ± 0.30 μg/mL of extract for α-amylase. The IC_50_ value registered in the case of acarbose was 2.09 ± 0.14 μg/mL for α-glucosidase and 4.49 ± 0.44 μg/mL for α-amylase. Acarbose showed a greater inhibitory effect towards α-glucosidase than α-amylase, the IC_50_ value obtained for α-glucosidase being 2 times lower than that of α-amylase. As shown in Table 4, the tested extract was more active towards α-glucosidase and α-amylase than the reference compound.

In other studies, Nile et al. [49] found that red onion solid waste extract exhibited good α-glucosidase inhibitory activity, with an IC_50_ of 48.6 ± 1.8 μg/mL, and with an IC_50_ of 10.1 ± 0.6, μg/mL for acarbose. IC_50_ values of 52.5 ± 1.1 μg/mL for α-amylase and 55.2 ± 1.3 μg/mL for α-glucosidase were reported for the yellow onion solid waste extract. Also, IC_50_ of 50.5 ± 1.2 μg/mL for α-amylase and 80.3 ± 1.7 μg/mL for α-glucosidase were reported for the positive control, acarbose [50]. Our results are higher than those of Oboh et al. [51] who reported that the aqueous extract of the purple onion exhibited weak inhibition on α-amylase (IC_50_ of 8.27 mg/mL), and α-glucosidase (IC_50_ of 4.50 mg/mL), respectively.

Kim et al. [52] investigated the in vitro inhibitory activity of the onion skins extract (Korean onion variety) against α-glucosidase and α-amylase. The extract presented IC_50_ values of 1.27 mg/mL for α-glucosidase, and higher than 3.00 mg/mL for α-amylase. Nickavar and Yousefian [53] reported the α-amylase inhibitory potential of a common onion *A. cepa* L. variety, which showed IC_50_ values of 16.36 mg/mL. The IC_50_ value of acarbose measured was 0.028 μg/mL. These results suggest that the red onion skins extract and its constituents may serve as a potential source of natural α-amylase and α-glucosidase inhibitors. The ability of the red onion skin extract to inhibit α-amylase and α-glucosidase can be credited to the numerous phytochemicals constituent present in the dry skins of the red onion such as flavonols [17,54] and anthocyanins [17,55], which have been shown to exhibit enzyme inhibitory activity.

In order to find alternative natural sources for obesity treatment, we evaluated the effect of the red onion skin extract on lipase activity (Table 4). The extract showed an IC_50_ value of 4.57 ± 0.86 μg/mL against the pancreatic lipase, while the orlistat presented an IC_50_ value of 3.18 ± 0.33 μg/mL. The IC_50_ value of the red onion skins extract was statistically similar to the standard inhibitor. Kim et al. [10] assessed the effects of onion (*Allium cepa*) skins extract on pancreatic lipase. The tested concentration (455 μg/mL) of the extract inhibited the pancreatic lipase, thus obtaining an IC_50_ value of 53.70 μg/mL, while the orlistat presented an IC_50_ value of 0.04 µg/mL. More recently, the lipase inhibitory potential of the yellow onion juice was also tested by Trisat et al. [56]. The sample inhibited the pancreatic lipase with an IC_50_ value of 9.5 ± 21.1 mg/mL. The pancreatic lipase inhibitory activity of the extract indicates a potential reduction of the digestion and absorption of dietary lipids.

The effect of the red onion skins extract was also measured against the pro-inflammatory enzyme LOX (Table 4). The IC_50_ value of the extract for LOX was 2.40 ± 0.71 μg/mL, while the positive control—quercetin, presented an IC_50_ value of 1.95 ± 0.20 μg/mL. According to the results obtained, the red onion skins extract possesses slightly lower LOX inhibitory activity compared to the standard used. In a study by Lesjak et al. [11] a common variety of yellow onion extract exhibited lower anti-inflammatory potential than the pure compound, but with a high IC_50_ value (100 µg/mL), suggesting the presence of active principles. Also, quercetin-3,4′-*O*-di-glucoside and quercetin aglycone exhibited high inhibitory potential towards LOX. Therefore, the authors concluded that these two compounds may contribute to the anti-inflammatory activity of *A. cepa* L. extract. Hence, the red onion skins extract was able to inhibit the LOX. These findings, together with the antioxidant activity observed in the extract may contribute to the reduction of inflammation.

**Figure 3:**
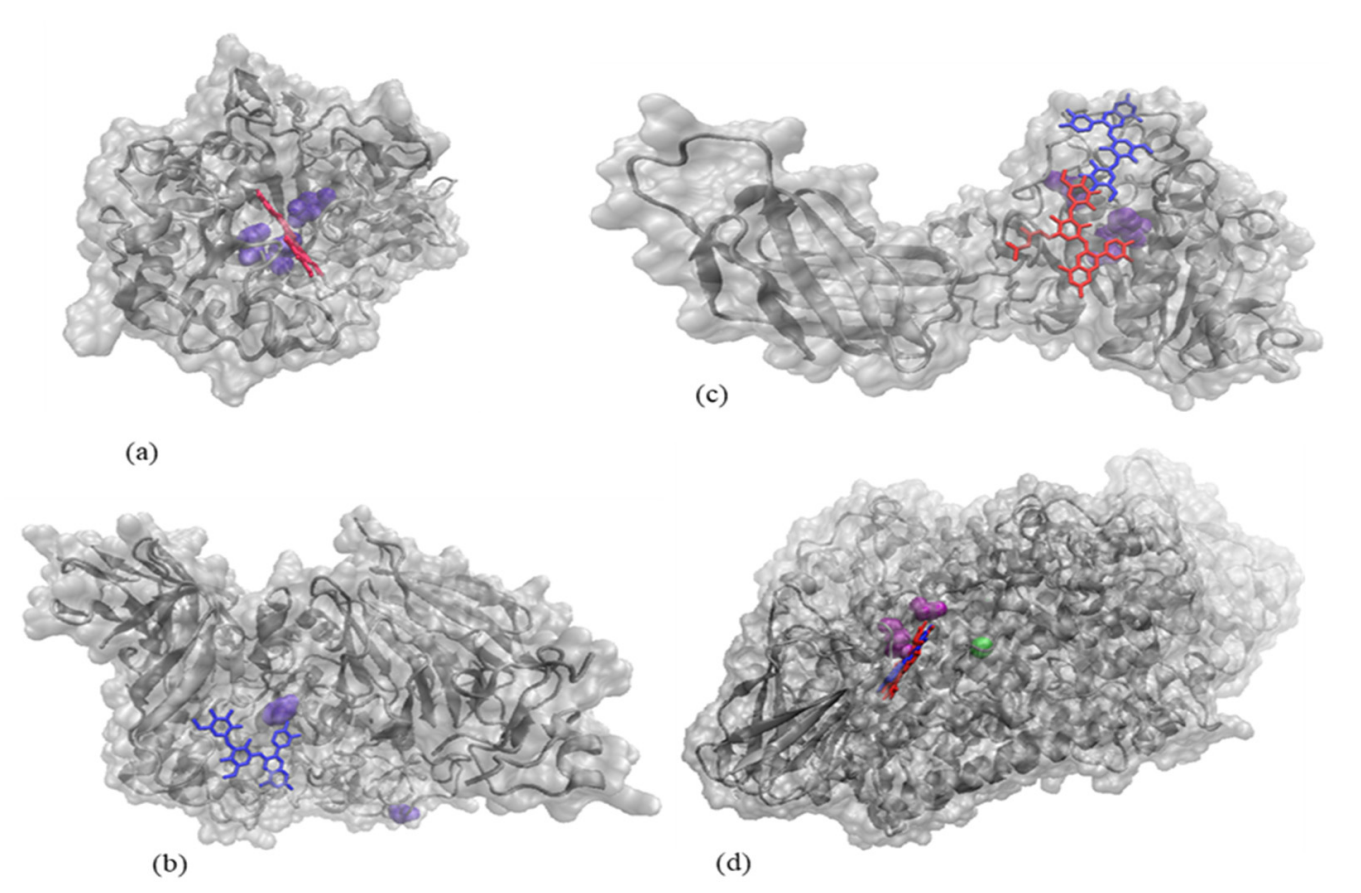
The results of the molecular docking tests showing the complexes formed by α-amylase (**a**) α-glucosidase (**b**), lipase (**c**), and LOX (**d**) shown in silver, with cyanidin 3-*O*-laminaribioside and/or cyanidin 3-*O*-(6″-malonyl-laminaribioside) represented in Licorice style in blue and red, respectively. The catalytic amino acids establishing contacts with the ligands are represented in purple in Van der Waals style. Images were prepared using VMD software [61].

Molecular docking tests were further employed to check if any of the two major anthocyanins identified in the red onion skins extract through HPLC directly binds to the active site of these enzymes, in such a manner to interfere with substrate recognition or its transformation. The in-depth analysis of the three top-scoring complexes showed that cyanidin 3-*O*-laminaribioside and/or cyanidin 3-*O*-(6″-malonyl-laminaribioside) might contribute to the inhibition of the activity of α-amylase, α-glucosidase, lipase, and LOX (Figure 3), therefore explaining the experimental findings presented in Table 4.

The activity of α-amylase appears to be potentially affected by the presence of cyanidin 3-*O*-(6″-malonyl-laminaribioside) which has a good affinity towards the active site of the enzyme, establishing contacts with the catalytic amino acids Asp^197^, Asp^300,^ and Glu^233^ [57]. The best fit predicted for the α-glucosidase—cyanidin 3-*O*-laminaribioside complex involves the direct contact of the ligand with the catalytic acid Asp^616^ [58]. In the case of lipase, both investigated ligands appear to be able to attach to the enzyme surface, near the hydrogen-bonded triad formed Ser^152^, Asp^176,^ and His^263^ [59]. Regarding LOX, both investigated ligands bind in the vicinity of Phe^177^ and Tyr^181^ located at one end of the active site cavity [60], being therefore potentially responsible for limiting the access of the substrate to the channel leading to the catalytic iron. Ligands binding near the active site of the four investigated enzymes might induce substantial local conformational changes, therefore, explaining the results of the experimental tests.

## 3. Materials and Methods

### 3.1. Materials

The red onion (*Allium cepa* L.) was purchased from a local marketplace (Galati, Romania) in July 2020. The skins were manually detached, washed with distilled water, and dried at 40 °C for 2 h in an oven (Stericell 111, MMM Medcenter, München, Germany) up to moisture content (MF-50 moisture analyzer, A&D Company, Tokyo, Japan) of 11.0%. The dried skins were then ground with a domestic grinder and stored in a hermetically sealed container at 4 °C until further analysis.

### 3.2. Chemicals

Gallic acid, 2,2-diphenyl-1-picrylhydrazyl (DPPH), methanol (HPLC grade), 6-hydroxy-2,5,7,8-tetramethylchromane-2-carboxylic acid (Trolox), sodium acetate solution, potassium chloride solution, α-glucosidase from *Saccharomyces cerevisiae* (≥10 units/mg protein), lipoxygenase from *Glycine max* (soybean) type I-B, 50,000 units/mg protein, α-amylase from porcine pancreas (type I-A, 700–1400 units/mg protein), pancreatin lipase (111.5 units/mg protein), linoleic acid (≥99%), p-nitrophenyl-α-d-glucopyranoside (≥97.5%), p-nitrophenyl palmitate (98%), starch solution, Triton X-100, Arabic gum, dinitrosalicylic acid (DNS), orlistat ≥ 98%, quercetin ≥ 95%, and acarbose ≥ 98% were acquired from Sigma Aldrich (Steinheim am Albuch, Germany). All chemicals and reagents utilized in the experiments were of analytical grade.

### 3.3. Extraction of Anthocyanins from the Red Onion Skins

The extraction of anthocyanins from the red onion skins was performed utilizing the UAE method, as described by Albishi et al. [62], with slight modifications. Therefore, 20 g of onion skins powder were mixed with 280 mL of aqueous ethanol 70% (*v/v*), acidified with glacial acetic acid (in a 6:1 ratio, % (*v/v*)). The mixture was sonicated for 20 min at 25 °C and 40 kHz (MRC Scientific 193 Instruments, Holon, Israel), followed by centrifugation at 14,000 rpm and 4 °C for 5 min (Universal 320R cooling ultracentrifuge, Hettich, Tuttlingen, Germany). In order to increase the extraction yield, three successive extractions were performed. Afterward, the supernatants were collected and concentrated to dryness at 40 °C, under reduced pressure (AVC 2-18, Christ, Osterode, Germany).

### 3.4. Extract Characterization

The extract was spectrophotometrically described in terms of TAC, TPC, and antioxidant activity, as described by Stoica et al. [25]. Thus, 2 mg/mL of extract diluted in ultrapure water were used for the analysis. The TAC was measured utilizing the pH-differential method and the results were expressed as mg cyanidin-3-*O*-glucoside equivalent (C3G)/g dry weight (DW). The TPC was analyzed using the Folin–Ciocâlteu method and the results were expressed as mg gallic acid equivalent (GAE)/g DW. The antioxidant activity of the UAE extract was assessed using the DPPH as free radical and the results were expressed as mM of Trolox equivalents (TE)/g DW. All phytochemicals were evaluated using a UV-VIS spectrophotometer with data analysis software (Libra S22, Biochrom, Cambridge, UK).

### 3.5. Chromatographic Analysis of Anthocyanins

The chromatographic profile of the anthocyanins extracted from red onion skins was achieved using a Thermo Finnigan Surveyor HPLC system coupled to a Diode-Array Detector and controlled by the Xcalibur software (Finnigan Surveyor LC, Thermo Scientific, Waltham, MA, USA). Over 4 g of extract, a volume of 4 mL of 10% formic acid solution, and a volume of 16 mL HPLC purity methanol were added to reach a total volume of 20 mL, followed by homogenization. Prior to the chromatographic analysis, in order to exclude the majority of compounds that could interfere with the separation and identification of the anthocyanins from the red onion skins’ extract, the sample was filtered through a C18 Sep-Pack cartridge (Cartridge-Waters, Milford, MA, USA). In order to separate and identify the red onion skins’ compounds, a Synergi 4u Fusion-RP 80A (150 × 4.6 mm, 4 μm) column was used, monitored at 520 nm, at an oven temperature of 25 °C. The elution was performed using a flow rate of 1 mL/min with a gradient of mobile phase A (methanol 100%) and mobile phase B (water/formic acid 10%). The following gradient was used for the samples: 0–20 min, 9–35% (A); 20–30 min, 35% (A); 30–40 min, 35–50% (A); and 40–55 min, 50–9% (A). The injection volume was 10 μL for the samples. The anthocyanins from red onion skins extract were identified and quantified based on the retention time and by comparison to the available standards and data reported in the literature by Donner et al. [27], and Sharif et al. [63].

### 3.6. Thermal Treatment

For the heat treatment experiments, 2 mL of extract in ultrapure water (2 mg/mL) at a pH of 3.2, (1 N HCl) were filed into screw-cap test glass tubes. The glass tubes were heated in the temperature range of 75–155 °C for 15 to 60 min, using a block heater (Stuart SBH200D, Stafford, UK). The parameters were picked taking into account the pasteurization and sterilization temperatures and times. After the thermal treatment, the samples were immediately cooled using an ice water bath to avoid further degradation. The experiment was performed in triplicate and the TACs and antioxidant activities of the thermally treated extracts were determined using the spectrophotometric methods described above.

### 3.7. Kinetic Analysis

The experimental data concerning the change in TAC and antioxidant activity over time were fitted to the first-order kinetics model (Equation (1)). The kinetic rate constant (k) of thermal degradation, the half-life value (t_1/2_), decimal reduction time (D), z-values, and activation energy (Ea) were calculated as described by Peron et al. [12].

C/C_0_ = exp (−kt) (1)

where C is the concentration of the parameter after time t to be estimated (TAC or antioxidant activity), C_0_ signifies the concentration of the parameter in the initial condition (at time 0), (mg/g DW), respectively, t is the thermal processing time, and k is the kinetic rate constant at temperature T.

### 3.8. Thermodynamic Parameters

The thermodynamic parameters of TAC and antioxidant activity degradation, including the activation enthalpy (ΔH, kJ/mol), Gibbs free energy (ΔG, kJ/mol), and activation entropy (ΔS, J·mol^−1^·K^−1^) at each temperature were calculated according to Qiu et al. [40].

### 3.9. Molecular Modeling Investigations on Anthocyanins Behavior at Thermal Treatment

The thermal-dependent behavior of the main anthocyanin molecules identified in the red onion skins extract by means of HLPC was further checked by means of a molecular modeling approach. The molecular models of cyanidin 3-*O*-laminaribioside and cyanidin 3-*O*-(6″-malonyl-laminaribioside) were built, optimized, and equilibrated using the molecular modeling software HyperChem release 8.0 (Hypercube, Inc., Waterloo, ON, Canada). Two geometry optimization algorithms, namely Steepest descent and Conjugate gradient, were used in sequence to minimize the potential energy of the models until reaching a root means square gradient of 0.0001 kcal/(Å·mol). Further molecular dynamics heating and equilibration steps at temperatures ranging from 75 to 155 °C, were carried out to simulate the thermal treatment and to check at a single molecule level any important changes occurring in the models.

### 3.10. In Vitro Enzymes Activity Inhibition

#### 3.10.1. α-Amylase Inhibition Assay

The α-amylase inhibition by the red onion skins extract was measured according to Costamagna et al. [64], with slight modifications. Briefly, a volume of 100 µL of extract solutions (0.5, 1, and 5 µg/mL concentrations of extract diluted in ultrapure water) was added to 100 µL of α-amylase solution (1 mg/mL in 0.1 M PBS, pH = 6.9). After 5 min of incubation at room temperature, 100 µL of 1% (*w/v*) starch solution in distilled water was added into the reaction mixture and incubated for another 20 min at 37 °C. Further, 200 µL of 0.04 M DNS reagent was added into the reaction mixture, followed by heating at 100 °C for 5 min in a thermostatic water bath (Digibath-2 BAD 4, Raypa Trade, Barcelona, Spain). Finally, the samples were diluted with 2 mL of distilled water and the absorbance was measured at 540 nm with a UV-VIS spectrophotometer. Acarbose was used as a standard inhibitor. The results of α-amylase inhibition activity were expressed in terms of IC50 value (μg/mL of extract). The IC50 value (μg/mL) represents the extract concentration at which 50% of the enzyme activity is inhibited and was graphically plotted using the logarithmic concentrations of the extract versus the inhibition percentages of the enzyme.

#### 3.10.2. α-Glucosidase Inhibition Assay

The α-glucosidase inhibitory activity of the red onion skins extract was also measured according to Costamagna et al. [64]. The reaction mixture contained 50 µL of α-glucosidase solution (1 mg/mL in 0.1 M PBS, pH = 6.9) and 50 µL extract solutions (0.5, 1, and 5 µg/mL). After the pre-incubation of the reaction mixture at room temperature for 5 min, the enzyme reaction started by adding 50 µL of 25 mM p-nitrophenyl a-d-glucopyranoside and 1.6 mL of 0.1 M PBS, pH = 6.9. The mixture was incubated for 15 min at 37 °C. Then, 800 µL of 0.2 M sodium carbonate was added. The absorbance was read at 405 nm with a UV-VIS spectrophotometer. Acarbose was used as a standard inhibitor. The inhibitory effect of the extract was expressed as IC50 (μg/mL) value.

#### 3.10.3. Lipase Inhibition Assay

The lipase inhibitory effect of the extract was also assayed according to Costamagna et al. [64]. In brief, a volume of 50 μL of pancreatin lipase solution (1 mg/mL in 0.1 M PBS, pH = 8.0) was mixed with 50 μL of red onion skins extract (0.5, 1, and 5 µg/mL) and kept at room temperature for 5 min. Then, 50 µL of the substrate, obtained with 0.01 M p-nitrophenyl palmitate (in 0.1 M PBS), 0.6% (*w/v*) Triton X-100, and 0.15% (*w/v*) Arabic gum was added and the solution was incubated at 37 °C for 20 min. More, the samples were diluted with 1 mL of 0.1 M PBS at pH = 8.0. The absorbance was read at 400 nm with a UV-VIS spectrophotometer. Orlistat was used as the standard inhibitor. The inhibitory effect of the extract was expressed as IC50 (μg/mL) value.

#### 3.10.4. Lipoxygenase Inhibition Assay

The biotest for the lipoxygenase (LOX) inhibition was performed according to Costamagna et al. [64], with slight modifications. Briefly, a volume of 50 µL LOX solution (1 mg/mL in 0.1 M PBS, pH = 9.0) was mixed with 50 µL of extract solution (0.5, 1, and 5 µg/mL) and pre-incubated on room temperature for 5 min. Then 50 μL of 0.05 mM linoleic acid dissolved in 0.1 M PBS (pH = 9.0) was added, and the mixture was incubated at 37 °C for 20 min. Afterward, the samples were diluted with 2 mL of 0.1 M PBS at pH = 9.0, and the absorbance was read at 234 nm with a UV-VIS spectrophotometer in quartz cuvettes. Quercetin was used as a standard inhibitor. The inhibitory effect of the extracts was expressed as the IC50 (μg/mL) value.

### 3.11. In Silico Testing the Anthocyanins Binding to Enzymes

Molecular docking tests were employed to identify the sites preferentially targeted by cyanidin 3-*O*-laminaribioside and cyanidin 3-*O*-(6″-malonyl-laminaribioside) when interacting with α-amylase, α-glucosidase, lipase, and LOX. After appropriate refinement, the three-dimensional models of the enzymes taken from the RCSB Protein Data Bank (PDB IDs 6Z8L [57], 5NN5 [58], 1N8S [65], 3O8Y [60] were used as receptors for the anthocyanins. The rigid docking procedure based on the molecular shape complementarity was done with the PatchDock algorithm [66]. The top three protein-ligand models scored based on the interaction energy values were further investigated for gathering insight on the potential mechanism responsible for enzymes inhibition.

### 3.12. Statistical Analysis

All experiments were performed in triplicate, and the results were expressed as mean values ± standard deviation. The differences between the samples were assessed by the Tukey test with the one-way analysis of variance (ANOVA) method for the data that followed the normal distribution and equal variances conditions. The parameters of the kinetic models and the Arrhenius equation were estimated using linear regression. Mathematical models were selected by comparing correlations coefficients.

## 4. Conclusions

The red onion skins extract exhibited high total phenolics and anthocyanins contents and high antioxidant activity, which can increase its popularity as a promising source of bioactive compounds. The chromatographic profile showed the presence of eight main anthocyanins, with the major ones being cyanidin 3-*O*-laminaribioside and cyanidin 3-*O*-(6″-malonyl-laminaribioside). The kinetics of the thermal degradation of total anthocyanins content and antioxidant activity were studied during heating at different temperatures (75–155 °C) and time (0–60 min). The results revealed that the degradation of both total anthocyanins content and antioxidant activity followed the first-order reaction kinetics, with the effect of temperature being adequately described by the Arrhenius model. The kinetic parameters revealed that the increase in temperature accelerated the degradation effect. Thus, higher stability of bioactive compounds was found at lower temperatures and shorter heating times during the thermal treatment. Moreover, the thermodynamic parameters confirmed the previous presumptions of irreversible and nonspontaneous reactions. The extract was also able to inhibit α-glucosidase, α-amylase, lipase, and lipoxygenase, enzymes associated with the metabolic syndrome and the inflammatory process. 

The results presented in this study concerning the red onion skins anthocyanins provide the necessary information that enables the valorization of these by-products in multiple potential applications. These applications could refer to several industrial uses as nutraceuticals, food preserving agents, and pharmaceuticals. With extraordinary bioactivity, the phytochemicals present in the onion skins extract can be further investigated for the formulation of functional food ingredients.

## Figures and Tables

**Table 1:** The extract phytochemical characterization.

Selected Phytochemical	Extract
TAC, mg C3G/g DW	2.75 ± 0.15
TPC, mg GAE/g DW	172.17 ± 3.01
Antioxidant activity, mM TE/g DW	436.25 ± 3.51

**Table 2:** The degradation kinetic parameters (degradation rate constant—k, correlation coefficient —R^2^, decimal reduction time—D, z-value, activation energy—Ea, and half-life values—t_1/2_) of the total anthocyanins content (TAC) and antioxidant activity from the red onion skins extract heat-treated at different temperatures.

Compounds	Temperature °C	k·10^−2^ (min^−1^)	R^2^	t_1/2_ (min)	D (min)
TAC	75	0.69 ± 0.08	0.96	100.32 ± 1.91	333.33 ± 6.85
	95	1.38 ± 0.21	0.96	50.16 ± 1.42	166.67 ± 4.68
	115	2.53 ± 0.83	0.98	27.36 ± 0.93	90.91 ± 3.45
	135	7.37 ± 1.33	0,98	9.41 ± 0.72	31.25 ± 2.06
	155	18.65 ± 2.36	0.98	3.72 ± 0.48	12.35 ± 1.08
	Ea (kJ·mol^−1^) = 50.77 ± 1.71 (R^2^ = 0.97) Z (°C) = 55.56 ± 2.91 (R^2^ = 0.98)				
Antioxidant activity	75	0.48 ± 0.27	0.92	143.32 ± 2.42	476.19 ± 10.31
	95	0.69 ± 0.42	0.93	100.33 ± 2.51	333.33 ± 9.57
	115	0.85 ± 0.64	0.93	81.34 ± 1.82	270.27 ± 8.67
	135	1.04 ± 0.75	0.98	66.88 ± 1.74	222.22 ± 7.48
	155	1.33 ± 0.84	0.99	51.89 ± 1.46	172.41 ± 6.12
	Ea (kJ·mol^−1^) = 15.13 ± 2.05 (R^2^ = 0.99) Z (°C) = 188.68 ± 5.11 (R^2^ = 0.98)				

**Table 3:** Thermodynamic parameters (ΔH, ΔG, and ΔS) of phytochemical degradation process in red onion skins extract.

Compounds	Temperature (K)	ΔH (kJ/mol)	ΔG (kJ/mol)	ΔS (J/(mol·K))
	348	47.88 ± 0.62	111.91 ± 1.37	−184.02 ± 2.18
	368	47.71 ± 0.28	116.40 ± 1.32	−186.65 ± 2.15
TAC	388	47.54 ± 0.30	120.94 ± 3.49	−189.16 ± 1.43
	408	47.38 ± 0.34	123.72 ± 2.14	−187.12 ± 1.33
	428	47.21 ± 0.27	126.65 ± 2.18	−185.61 ± 1.56
	348	12.24 ± 0.16	112.95 ± 3.53	−289.39 ± 1.41
	368	12.07 ± 0.23	118.52 ± 1.41	−289.25 ± 0.92
Antioxidant activity	388	11.91 ± 0.15	124.45 ± 2.83	−290.07 ± 0.71
	408	11.74 ± 0.28	130.37 ± 2.57	−290.77 ± 0.87
	428	11.57 ± 0.40	136.03 ± 2.12	−290.79 ± 1.06

**Table 4:** The enzyme inhibition capacity (IC_50_ values; μg/mL) of red onion skins extract in α-amylase, α-glucosidase, lipase, and LOX enzymes.

Sample	IC_50_ (μg/mL Extract)			
	α-Amylase	α-Glucosidase	Lipase	LOX
Extract	1.02 ± 0.30a	0.57 ± 0.16a	4.57 ± 0.86a	2.40 ± 0.71a
Acarbose	4.49 ± 0.44b	2.09 ± 0.14b	-	-
Orlistat	-	-	3.18 ± 0.33a	-
Quercetin	-	-	-	1.95 ± 0.20a

Measurements are expressed as mean ± SD of triplicates. Values (mean ± SD) from a column that shares the same letter are not significantly different (*p* > 0.05).

## Data Availability

The data that support the findings of this study are available from the corresponding author, G.R., upon reasonable request.
